# Effects of autotoxicity and allelopathy on seed germination and seedling growth in *Medicago truncatula*

**DOI:** 10.3389/fpls.2022.908426

**Published:** 2022-07-15

**Authors:** Chang Wang, Zhe Liu, Zicheng Wang, Wenhui Pang, Long Zhang, Zhaozhu Wen, Yiran Zhao, Juan Sun, Zeng-Yu Wang, Chao Yang

**Affiliations:** Key Laboratory of National Forestry and Grassland Administration on Grassland Resources and Ecology in the Yellow River Delta, College of Grassland Science, Qingdao Agricultural University, Qingdao, China

**Keywords:** *Medicago truncatula*, autotoxicity, forage crop, model plant, *Medicago sativa*

## Abstract

Autotoxicity is a form of intraspecific allelopathy, in which a plant species inhibits the establishment or growth of the same species through the release of toxic chemical compounds into the environment. The phenomenon of autotoxicity in crops is best traced in alfalfa (*Medicago sativa*). A close relative of alfalfa, *M. truncatula,* has been developed into an excellent model species for leguminous plants. However, it is not known whether *M. truncatula* has autotoxicity. In this study, *M. truncatula* root exudates showed a negative impact on the growth of *M. truncatula* seedlings, indicating autotoxicity. Detailed analyses with plant extracts from *M. truncatula* and alfalfa revealed varying degrees of suppression effects in the two species. The extracts negatively affected seed germination potential, germination rate, radicle length, hypocotyl length, synthetic allelopathic effect index, plant height, root growth, fresh weight, dry weight, net photosynthetic rate, transpiration rate, and stomatal conductance in both *M. truncatula* and alfalfa. The results demonstrated that autotoxicity and allelopathic effects exist in *M. truncatula*. This opens up a new way to use *M. truncatula* as a model species to carry out in-depth studies of autotoxicity and allelopathy to elucidate biochemical pathways of allelochemicals and molecular networks controlling biosynthesis of the chemicals.

## Introduction

Autotoxicity is a phenomenon in which a given plant species releases specific chemical substances to the environment to directly or indirectly affects the germination or growth of adjacent or next crop of the same species (Miller, 1996). Allelopathy is the suppression of growth of one plant species by another due to the release of toxic substances. Autotoxicity is a kind of intraspecific allelopathy (Chon et al., 2006). Autotoxicity has been observed in grasslands, fields, forests, orchards, etc., where it causes a number of ecological and economic complications such as a decline in crop yield, continuous cropping obstacles, regeneration failure of forests, and replant problem in orchards (Chon et al., 2006; Singh et al., 2010).

In crops, autotoxicity is best traced in alfalfa (*Medicago sativa*; Singh et al., 2010). Alfalfa is a widely grown high-quality forage crop in the world, it is the 4th most valuable crop in the United States (Du et al., 2021). It is known that due to autotoxicity, alfalfa cannot be interseeded into alfalfa field to thicken stands, and seeding alfalfa immediately after a terminated alfalfa stand without a significant time lapse will result in failed establishment. As a general rule, it is recommended to allow at least 1-year interval between terminating an old alfalfa stand and re-seeding a new stand. There have been a number of studies on alfalfa autotoxicity and some allelochemicals have been identified (Chon et al., 2003, 2006; Singh et al., 2010; Ghimire et al., 2019; Zhang et al., 2021a); however, like other species with autotoxic effects, the biochemical and molecular basis of autotoxicity has never been well explained (Cheng and Cheng, 2015). This is mainly because of the lack of an effective model system to carry out in-depth studies of autotoxicity and allelopathy. Species with large autotoxicity or allelopathy effects often have complex genetic and reproductive systems. For example, alfalfa is an outcrossing and tetraploid species with complex genomes. This makes it extremely difficult to conduct detailed mechanistic research. Thus, there is an urgent need of a model system to study autotoxicity and allelopathy.

In recent years, a close relative of alfalfa, *M. truncatula*, has been developed into a model legume plant (Tadege et al., 2008; Kang et al., 2016; Wolabu et al., 2020; Chai et al., 2021). This is mainly because *M. truncatula* is a selfing, diploid species with a small genome and relatively short life cycle (Tang et al., 2014; Kang et al., 2016). This model system has been widely adopted to study different traits, such as compound leaf development, nitrogen fixation, and biomass yield (Kang et al., 2016; Zhou et al., 2019; Mergaert et al., 2020; Chai et al., 2021). However, it is not known whether *M. truncatula* can be used as a suitable system to study autotoxicity and allelopathy.

The objective of this study was to determine whether *M. truncatula* has autotoxicity and allelopathy. By analyzing the effects of root exudates and plant extracts on germination and growth of *M. truncatula* and alfalfa, this study clearly demonstrates, for the first time, that autotoxicity and allelopathy exist in *M. truncatula.* Therefore, *M. truncatula* can be used as an effective model system to study the biochemical and molecular basis of autotoxicity and allelopathy.

## Materials and methods

### Plant materials

*Medicago truncatula* ecotype R108 and alfalfa variety Sanditi were used in this study. *M. truncatula* and alfalfa were planted in an artificial climate chamber under the condition of light/dark (16/8 h, 23/18°C) and humidity of 40%.

### Collection and activity identification of root exudates of *Medicago truncatula*

Sterilized *M. truncatula* seeds were lightly sanded with sandpaper and germinated in a Petri dish with two layers of moist filter paper. After 3 days, 20 germinated seedlings were placed in each hydroponic containers filled with 700 ml of distilled water. Distilled water was periodically added to the container to ensure the volume remain unchanged. The hydroponic *M. truncatula* seedlings were grown for 21 days under the condition of light/dark (16/8 h, 23/18°C) and humidity of 40%. The aqueous solution in each container was collected. The mixed solution was first filtered with 4 layers of gauze, and then the root exudates were mixed with distilled water. The volume of the root exudates accounted for 0, 20, 40, 60, 80, and 100% of the total volume, respectively. For the treatment with root exudates, 20 seedlings were planted in each container with four replicates. After 21 days, plants were harvested from each container to measure the fresh weight and dry weight.

### Extraction of autotoxic and allelopathic substances from *Medicago truncatula* and alfalfa

The whole plants of *M. truncatula* and alfalfa at the budding and early flowering stages were collected in mesh bags, and placed in a box for drying at 37°C. The dried plants were cut into 1–2 cm pieces, ground to powder with a vibrating ultra-fine grinder, and the powder was collected and stored in a sealed bag at 4°C in the dark for future use. *M. truncatula* and alfalfa powder samples (25 g) were weighed and placed in a 1,000 ml conical flask, 500 ml of ultrapure water was added, shaken and soaked, sealed with sealing film, and sonicated for 15 min to improve the extraction efficiency. The conical flask was wrapped with tin foil, and the samples in the conical flask were shaken and mixed at regular intervals. The solution obtained after leaching for 24 h was first filtered with four layers of gauze, and then filtered through a Buchner funnel and a layer of filter paper using a vacuum pump, then the extracts were collected. The above steps were repeated three times for the remaining samples, and the extracts from the last four times were mixed, and the solvent was removed by vacuum rotary evaporation at 40°C to obtain the extracts.

### Determination of biological activities of *Medicago truncatula* and alfalfa extracts

#### Germination test of *Medicago truncatula* and alfalfa seeds

The extracts of *M. truncatula* and alfalfa were dissolved in ultrapure water and the volume was made up to 1,000 ml, and the concentration 25 mg/ml (that is, the extract containing 25 mg of powder sample in 1 ml aqueous solution) was prepared as a stock solution and stored at 4°C in the dark. The stock solution was diluted and formulated into 2, 4, 6, 8, and 10 mg/ml extracts, and distilled water was used for the control group. Two layers of filter paper were placed in a Petri dish (9 cm in diameter), and 5 ml of distilled water or extracts of different concentrations was added, respectively, and three replicates were set for each treatment. Seeds of the *M. truncatula* were sanded by sandpaper to break physical dormancy, and 25 *M. truncatula* seeds and 50 alfalfa seeds that had been sterilized and uniform in size were put into the Petri dishes with different treatments, respectively. The dishes were sealed with sealing film to prevent water loss. The culture dishes were placed in an incubator, and the culture conditions were dark at 23°C for the first 3 days, and then incubated for 4 days under the conditions of 16 h light at 25°C and 8 h dark at 23°C. The number of germinated seeds was counted every 24 h. Germination is counted when the radicle broke through the seed coat and reached 2 mm in length. The germination potential of the seeds was measured after 3 days, and the germination rate of the seeds was measured after 7 days. Five *M. truncatula* and alfalfa seedlings from each Petri dish were randomly selected to measure the radicle length (cm) and hypocotyl length (cm), and response index (RI) and synthetic allelopathic effect index (SE) were calculated (Zhang et al., 2019; Wang et al., 2020). The calculation methods for each indicator are as follows:

1. Germination potential = the number of normal germinated seeds within 3 days/the number of tested seeds × 100%.
2. Germination rate = the number of normal germinated seeds within 7 days/the number of tested seeds × 100%.
3. Response index: RI = 1−C/T (T ≥ C), RI = T/C−1 (T < C). T is the data of the treatment group, C is the data of the control group, RI < 0 represents an inhibitory effect, and RI > 0 represents a promoting effect.
4. Synthetic allelopathic effect index: SE = (RI1 + RI2 + … + RIn)/n.

#### Growth test of *Medicago truncatula* and alfalfa seedlings

Based on the results of seed germination experiments, the extracts with a concentration of 6 mg/ml were selected for the seedling growth test. The sterilized *M. truncatula* and alfalfa seeds were germinated, and when the seedlings grew to the two-leaf stage, they were transferred to a hydroponic box with a volume of 700 ml, and 15 seedlings were planted in each box. The control group was supplemented with Hoagland solution, and the treatment group was supplemented with Hoagland solution containing 6 mg/ml extracts of *M. truncatula* and alfalfa, respectively. Each treatment was repeated three times, and the hydroponic box was placed in an artificial climate room. Distilled water was added to maintain the solution volume. After growing for 14 days, the relevant traits and parameters of the *M. truncatula* and alfalfa seedlings with different treatments were determined. Plant height, dry weight, and fresh weight were measured. The LI-6800 portable photosynthesis system (Lincoln, NE, United States) was used to measure transpiration rate, net photosynthetic rate, intercellular CO_2_ concentration, and stomatal conductance. The root systems of the plants were scanned with a ScanMaker i800 plus flatbed scanner, and the scan results were analyzed by LA-S plant root analysis system (Wan Shen, Hangzhou, China) to obtain the total root length, root area and root volume.

### Statistical analysis

The experimental data were statistically analyzed using SPSS 26.0 software, and Origin 2019b was used for graphing.

## Results

### Effects of root exudates of *Medicago truncatula* on its fresh weight and dry weight

**Figure 1:**
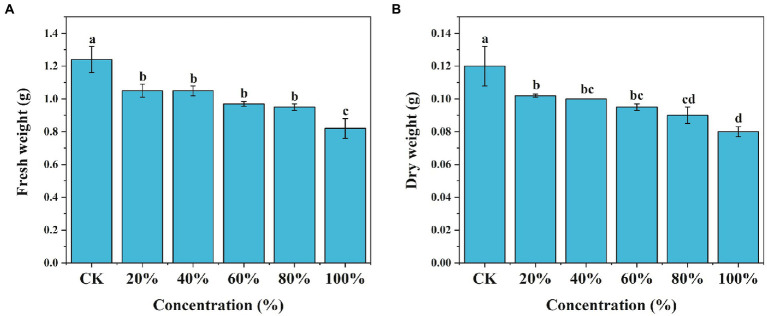
Effects of root exudates of *M. truncatula* on its fresh and dry weight. **(A)** *M. truncatula* fresh weight, **(B)** *M. truncatula* dry weight. Values represent means±SD of four biological replicates. Different letters above the bars indicate a significant difference (*p* < 0.05).

Root exudates collected from *M. truncatula* were used to test the growth response of *M. truncatula* seedlings. With the increase of the concentrations of the exudates, the inhibitory effects on the fresh weight and dry weight of *M. truncatula* gradually increased (Figures 1A,B). At 20% concentration, root exudates started to show a significant inhibitory effect on the fresh weight and dry weight of *M. truncatula*. Root exudates at concentrations of 20, 40, 60, 80, and 100% reduced the fresh weight of *M. truncatula* by 15, 15.2, 22.5, 23.5, and 31.7%, respectively (Figure 1A). Similarly, root exudates at concentrations of 20, 40, 60, 80, and 100% reduced the dry weight of *M. truncatula* by 14.8, 16.8, 20.3, 24.9, and 32.9%, respectively (Figure 1B).

### Effects of plant extracts on seed germination and seedling growth of *Medicago truncatula* and alfalfa

#### Effects of *Medicago truncatula* extracts on germination potential and germination rate of *Medicago truncatula* and alfalfa

**Figure 2:**
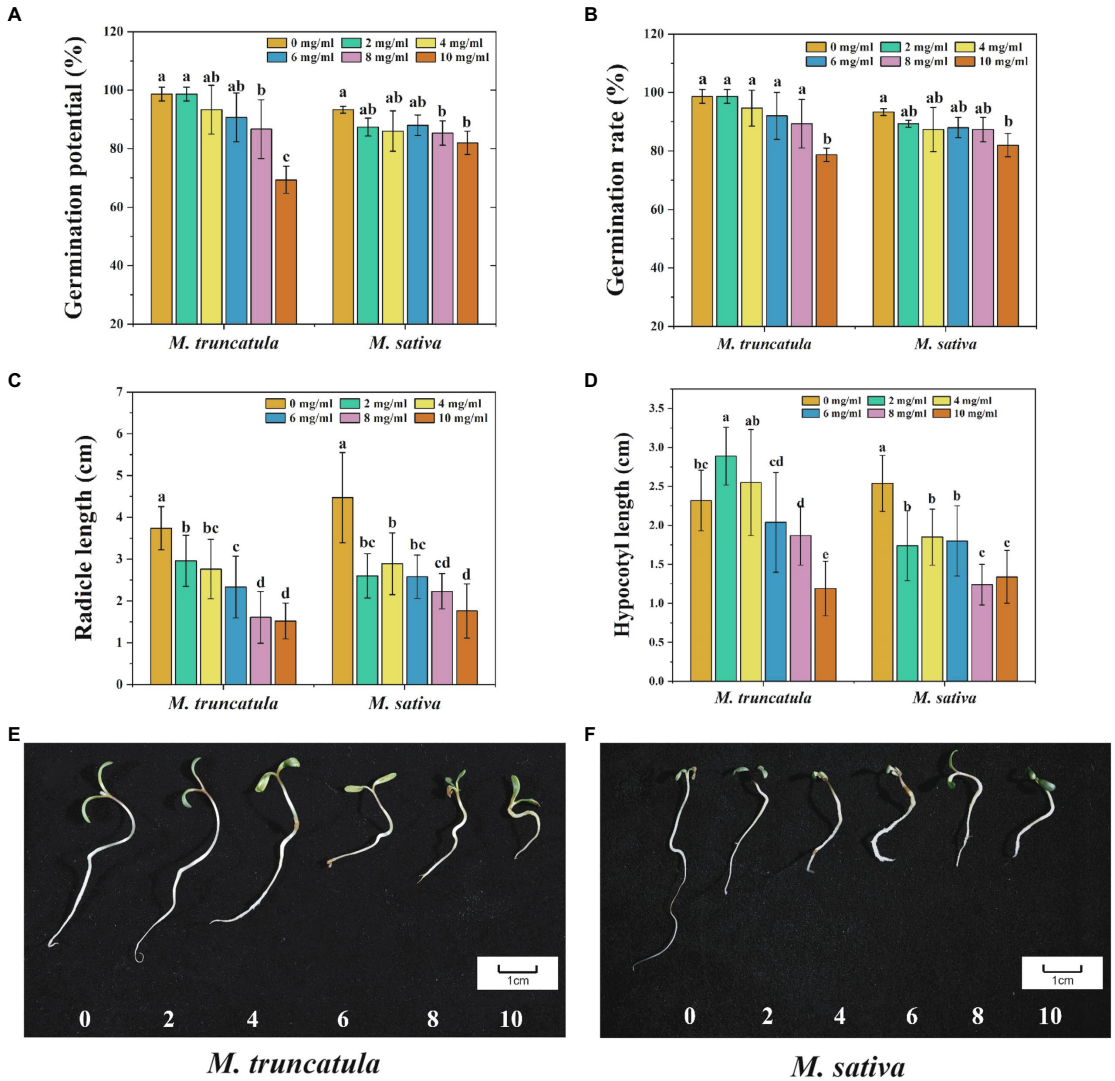
Effects of *M. truncatula* extracts on seed germination and seedling growth of *M. truncatula* and alfalfa. **(A)** Germination potential; **(B)** germination rate; **(C)** radicle length; and **(D)** hypocotyl length; **(E)**: *M. truncatula* seedling growth; **(F)**: alfalfa seedling growth. Values represent means±SD of three biological replicates. Different letters above the bars indicate a significant difference (*p* < 0.05).

The autotoxic and allelopathic substances from *M. truncatula* and alfalfa were extracted. At low concentrations (2–6 mg/ml), the extracts of *M. truncatula* had no significant impact on the germination potential and germination rate of *M. truncatula* and alfalfa (Figures 2A,B). When the concentration of the extracts reached 8 mg/ml and 10 mg/ml, the germination potential of both species was significantly reduced by 12 and 29.34% (*M. truncatula*) and 8 and 11.3% (alfalfa), respectively (Figure 2A). When the concentration of the extracts reached 10 mg/ml, the germination rate of *M. truncatula* and alfalfa decreased by 20 and 11.33%, respectively (Figure 2B).

#### Effects of *Medicago truncatula* extracts on the growth of radicle and hypocotyl of *Medicago truncatula* and alfalfa

During the process of seed germination, different concentrations of *M. truncatula* extracts all showed a significant inhibitory effect on the growth of radicles. The higher the concentration of the extracts, the stronger the corresponding inhibitory effect (Figure 2C). From low to high concentration, the inhibitory effects on radicle length of *M. truncatula* were 20.86, 26.2, 37.7, 56.95, and 59.36%, respectively, and the inhibitory effects on alfalfa radicle length were 41.83, 35.35, 42.28, 50.11, and 60.63%, respectively (Figure 2C).

The effects of *M. truncatula* extracts on the growth of the hypocotyl were different in *M. truncatula* and alfalfa (Figure 2D). When the concentration of the extracts was at 2 mg/ml, the growth of *M. truncatula* hypocotyl was significantly promoted by 24.57% (Figure 2D). Concentrations at 4 mg/ml and 6 mg/ml had no significant impact on the growth of *M. truncatula* hypocotyls. When the concentration reached 8 mg/ml and 10 mg/ml, the growth of *M. truncatula* hypocotyl was significantly inhibited by 19.4 and 48.71%, respectively. On the other side, the extracts at all concentrations had inhibitory effects on the growth of hypocotyls in alfalfa, and the strongest inhibitory effect was 51.18% when the concentration was at 8 mg/ml (Figure 2D).

#### Effects of *Medicago truncatula* extracts on synthetic allelopathic effect index during the germination of *Medicago truncatula* and alfalfa

Synthetic allelopathic effect index indicated that *M. truncatula* extracts had stress effects on the germination of *M. truncatula* and alfalfa, and the stress intensity increased with the increase of the concentration (Table 1). At low concentrations (2 mg/ml, 4 mg/ml), *M. truncatula* was less affected than alfalfa (Table 1). When the concentration reached 10 mg/ml, and the inhibitory effects on *M. truncatula* and alfalfa were 47.3 and 43%, respectively (Table 1; Figures 2E,F).

**Table 1:** Effects of *M. truncatula* extracts on synthetic allelopathic effect index (SE).

Concentration(mg/ml)	0	2	4	6	8	10
*M. truncatula*	0	−3.04%	−10.06%	−21.75%	−32.81%	−47.28%
*M. sativa*	0	−20.74%	−21.54%	−27.53%	−38.09%	−42.95%

#### Effects of alfalfa extracts on germination potential and germination rate of *Medicago truncatula* and alfalfa

**Figure 3:**
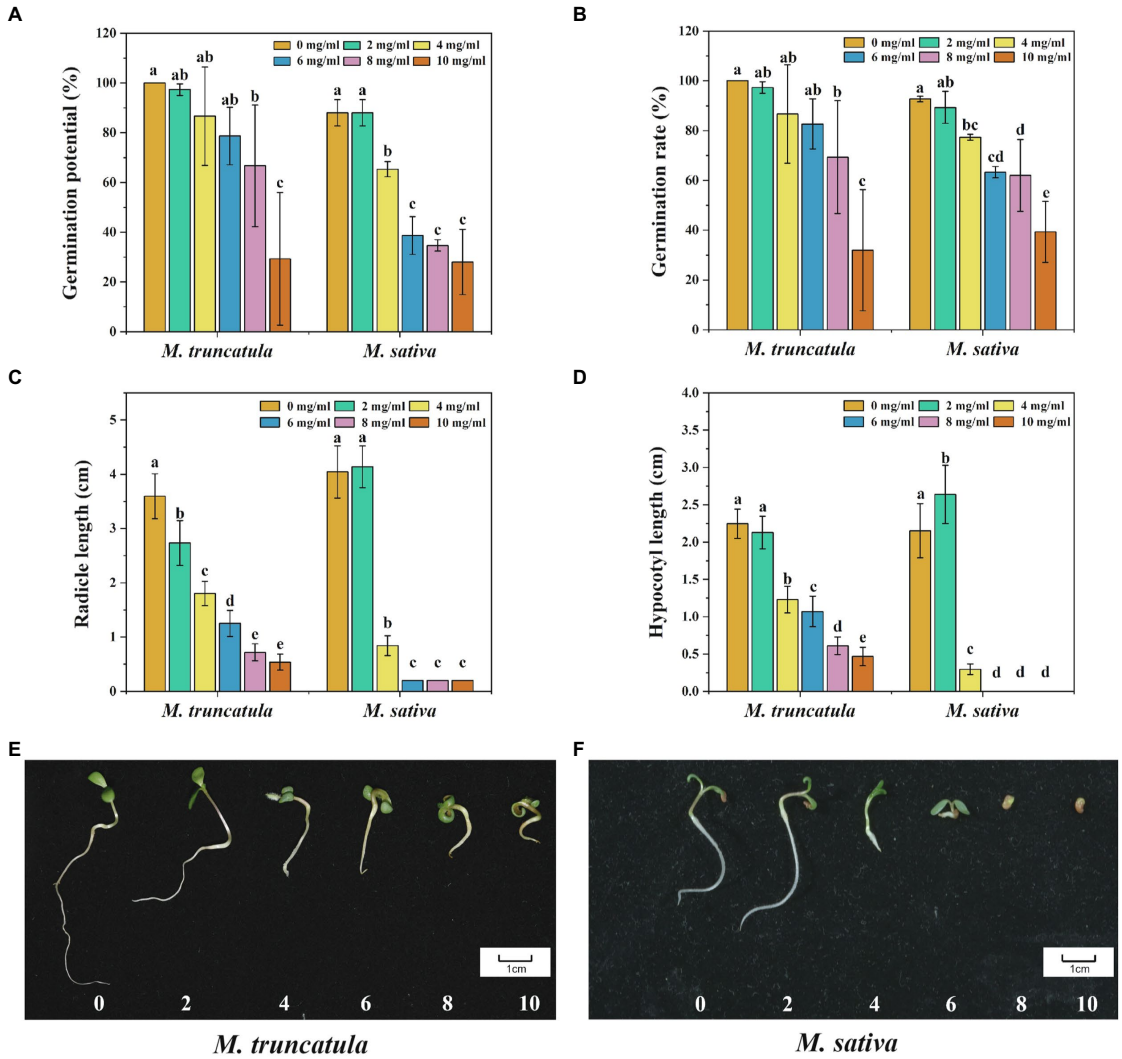
Effects of alfalfa extracts on seed germination and seedling growth of *M. truncatula* and alfalfa. **(A)** Germination potential; **(B)** germination rate; **(C)** radicle length; **(D)** hypocotyl length; **(E)** *M. truncatula* seedling growth; and **(F)** alfalfa seedling growth. Values represent means±SD of three biological replicates. Different letters above the bars indicate a significant difference (*p* < 0.05).

When the concentration of the extracts was at 8 mg/ml, it had a significant inhibitory effect on the germination potential and germination rate of *M. truncatula*, which decreased by 33.33 and 31.67%, respectively (Figures 3A,B). When the concentration of the extract was 4 mg/ml, it had a significant inhibitory effect on the germination potential and germination rate of alfalfa, which decreased by 12.67 and 15.34%, respectively. When the concentration was at 10 mg/ml, the germination potential of *M. truncatula* and alfalfa dropped below 30%, and the germination rate dropped below 40% (Figures 3A,B).

#### Effects of alfalfa extracts on the growth of radicle and hypocotyl of *Medicago truncatula* and alfalfa

All concentrations tested showed significant inhibitory effects on the growth of *M. truncatula* radicle (Figure 3C). With the increase of extract concentrations, the inhibitory effects on the growth of radicles were stronger, with reductions by 23.96, 49.82, 65.18, 80.01, and 85% at concentrations of 2, 4, 6, 8, and 10 mg/ml, respectively (Figure 3C). Regarding the growth of alfalfa radicle, no significant impact was observed when the concentration of the extracts was at 2 mg/ml. Drastic reduction (79.19%) in the growth of alfalfa radicle was found when the concentration of the extracts was at 4 mg/ml (Figure 3C). At the concentrations of 6, 8, and 10 mg/ml, the alfalfa seed radicle broke through the seed coat for 2 mm and then stopped growing (Figure 3C).

The effects of alfalfa extracts on the growth of *M. truncatula* hypocotyl were similar to that of radicle. When the concentration was 4 mg/ml, it began to produce a significant inhibitory effect on the growth of hypocotyl of *M. truncatula* (Figure 3D). The inhibitory effects were 45.22, 52.31, 72.77, and 79.19% at concentrations of 4, 6, 8, and 10 mg/ml, respectively. However, regarding hypocotyl growth of alfalfa, at 2 mg/ml, the extracts showed a promoting effect, making it elongated by 22.66% more than the control. At the concentration of 4 mg/ml, the alfalfa hypocotyl length was reduced by 86.27%. At concentrations of 6, 8, and 10 mg/ml, no hypocotyl growth of alfalfa seed was observed (Figure 3D).

#### Effects of alfalfa extracts on synthetic allelopathic effect index during the germination of *Medicago truncatula* and alfalfa

Synthetic allelopathic effect index showed that different concentrations of alfalfa extracts had various degrees of effects on the germination of *M. truncatula* and alfalfa. When the concentration of the extracts was 2 mg/ml, the inhibitory effect on *M. truncatula* was greater than alfalfa (Table 2). Starting from 4 mg/ml, the inhibitory effects on *M. truncatula* were less than alfalfa (Table 2). As shown in Figures 3E,F, when the concentrations were at 8 and 10 mg/ml, seed germination and growth were severely affected.

**Table 2:** Effects of alfalfa extracts on synthetic allelopathic effect index (SE).

Concentration(mg/ml)	0	2	4	6	8	10
*M. truncatula*	0	−11.28%	−32.26%	−48.18%	−62.25%	−79.63%
*M. sativa*	0	−1.33%	−57.49%	−73.61%	−75.58%	−83.56%

## Effects of the plants extracts on the growth of *Medicago truncatula* and alfalfa seedlings

### Effects of different plant extracts on plant height

**Figure 4:**
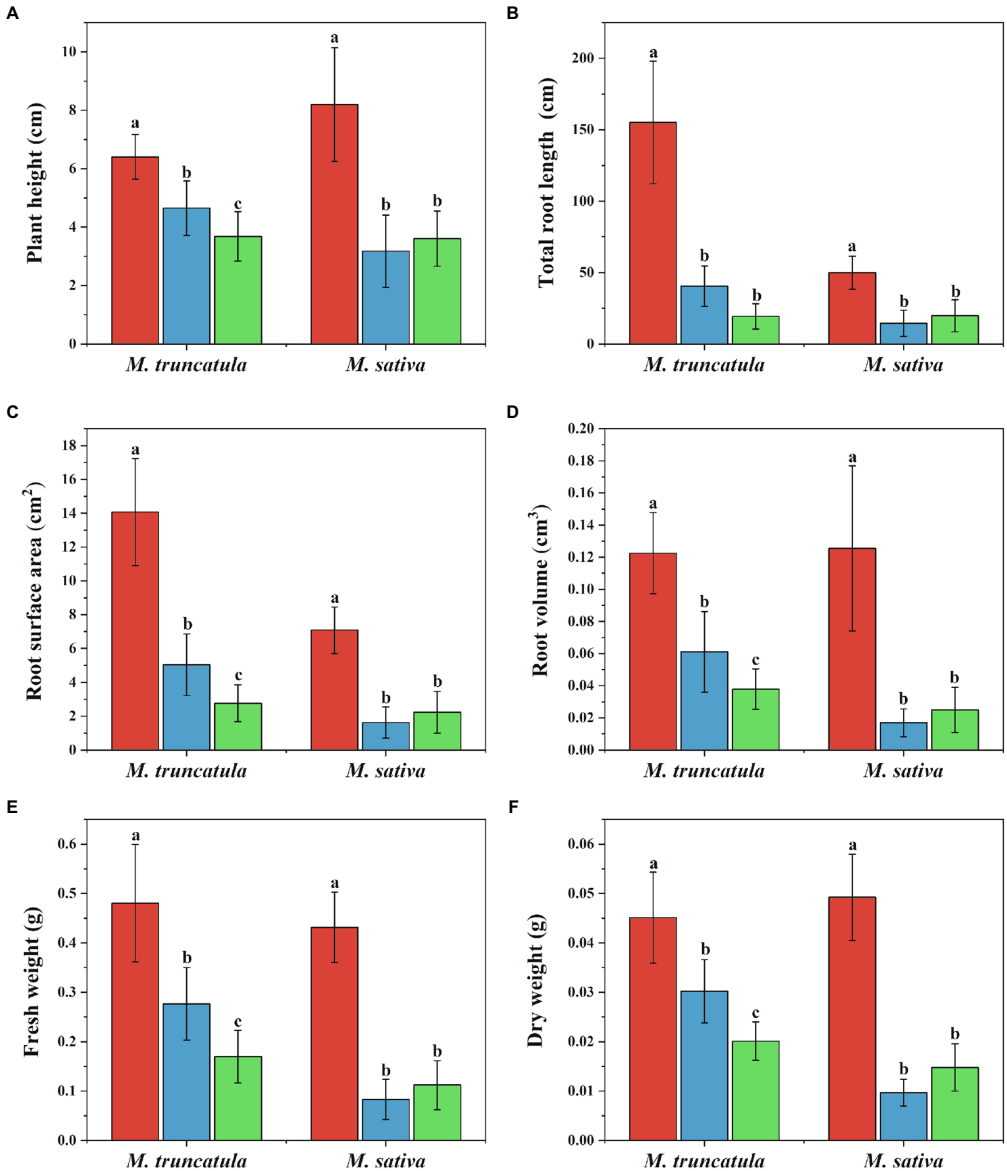
Effects of different plant extracts on plant phenotype during the growth of seedlings. **(A)** Plant height; **(B)** total root length; **(C)** root surface area; **(D)** root volume; **(E)** fresh weight; **(F)** dry weight. Values represent means±SD of seven biological replicates. Different letters above the bars indicate significant difference (*p* < 0.05). 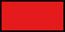 Control 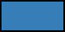 *M. truncatula* extracts 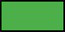 Alfalfa extracts.

**Figure 5:**
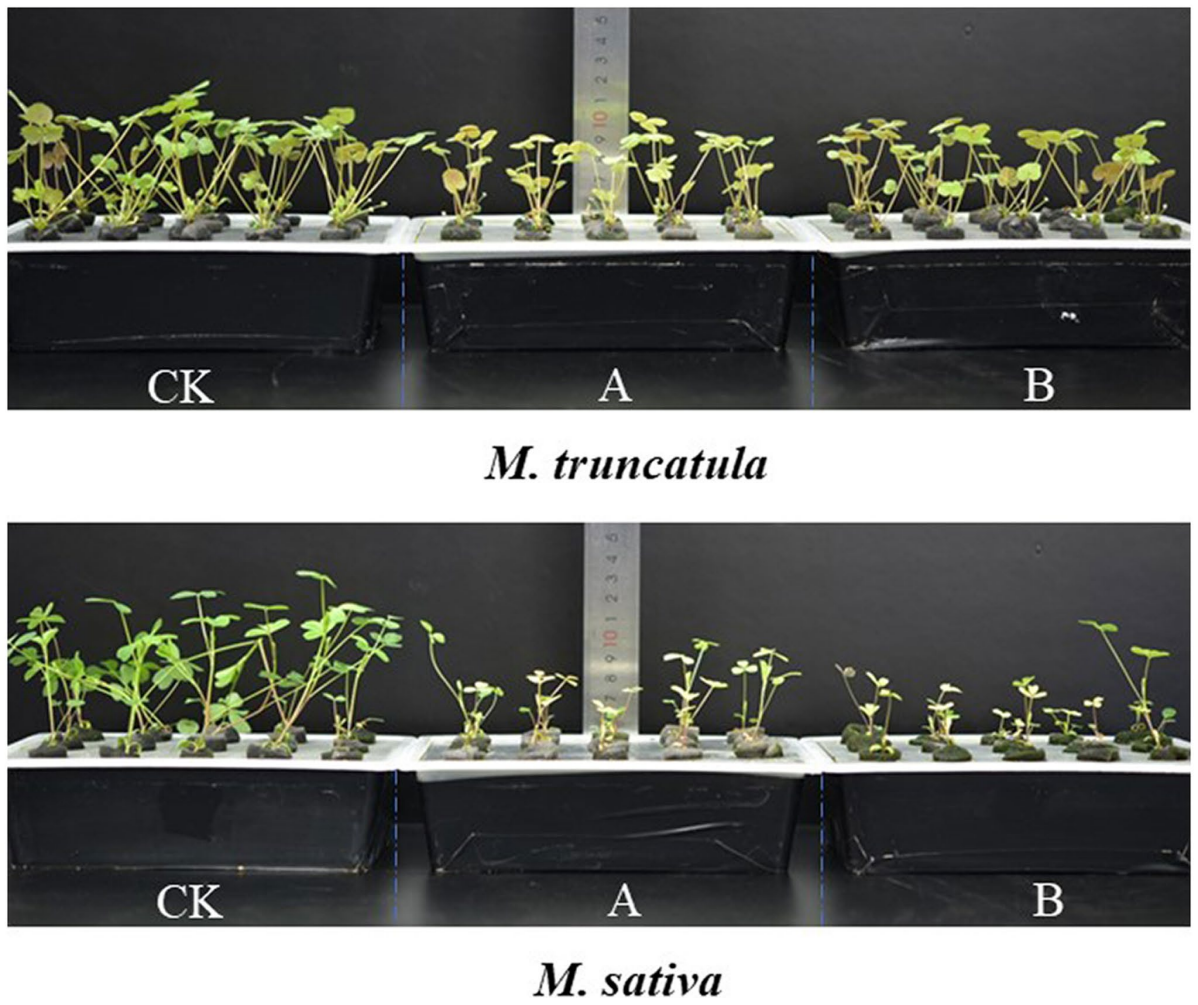
Plant height of *M. truncatula* and alfalfa 14 d after adding different plant extracts. CK: Control; A: alfalfa extracts and B: *M. truncatula* extracts.

Based on the results of above experiments (2.2), extracts concentration at 6 mg/ml was selected and tested for their impact on seedling growth. Extracts of *M. truncatula* and alfalfa had a stress effect on the height of the two species (Figure 4). Compared to the control, *M. truncatula* and alfalfa extracts reduced the height of *M. truncatula* by 27.42 and 42.54% and alfalfa by 61.25 and 56.05%, respectively (Figures 4A, 5).

### Effects of different plant extracts on root growth

**Figure 6:**
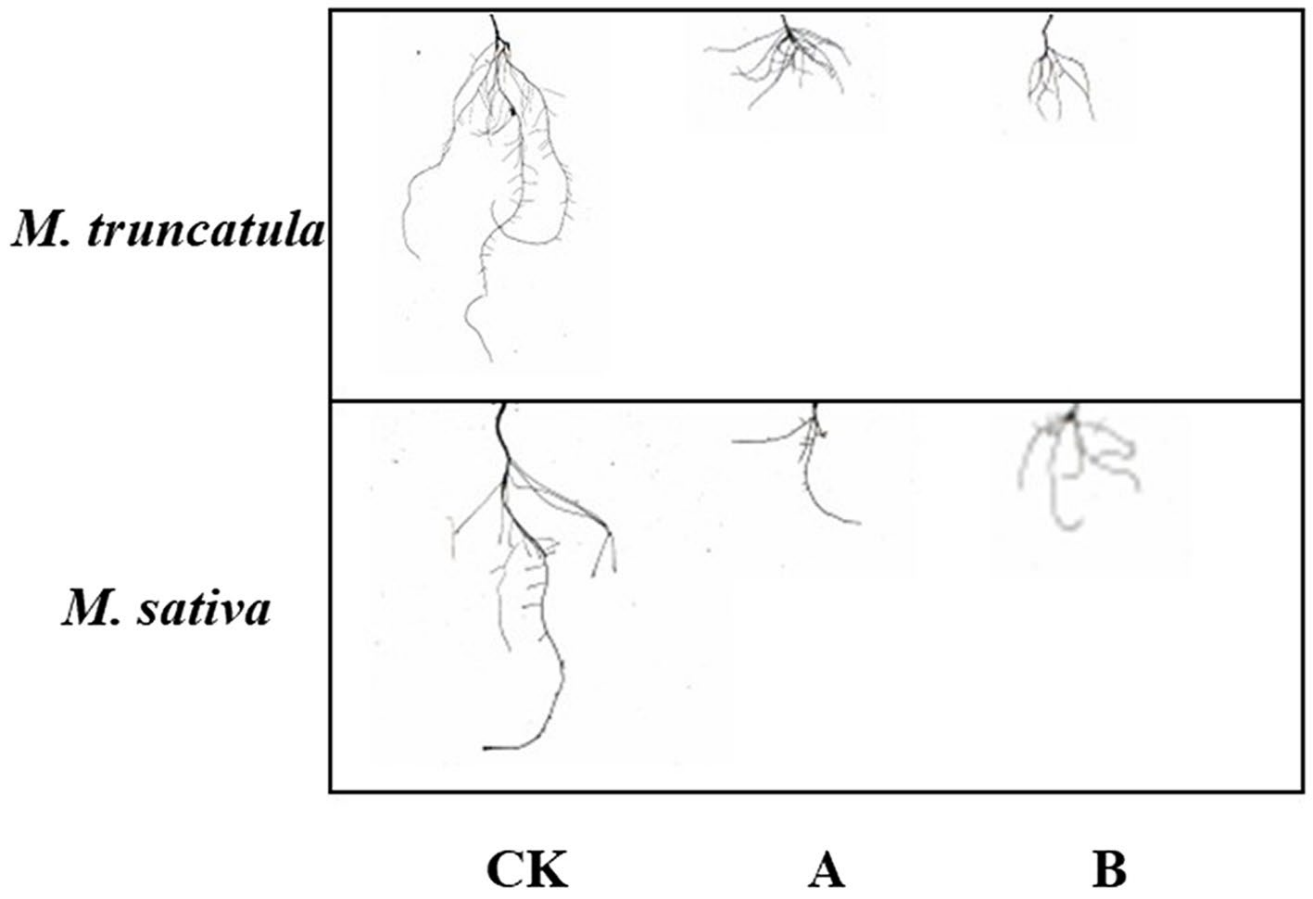
Effects of two extracts on root morphology of *M. truncatula* and alfalfa. CK: Control; A: *M. truncatula* extracts and B: alfalfa extracts.

Three indicators, total root length, root surface area, and root volume, were used to reflect the effects of the two plant extracts on root growth (Figures 4B–D, 6). Both plant extracts exerted stress on the root growth of *M. truncatula* and alfalfa, and the total root length, root surface area, and root volume of the treated group were significantly smaller than those of the control group. After being treated with 6 mg/ml *M. truncatula* and alfalfa extracts, the root length of *M. truncatula* decreased by 73.92 and 87.58%, the root surface area decreased by 64.17 and 80.42%, and the root volume decreased by 50.16 and 69.12%, respectively; the root length of alfalfa decreased by 71.08 and 60.46%, the root surface area decreased by 77.1 and 68.56%, and the root volume decreased by 86.47 and 80.17%, respectively (Figures 4B–D, 6).

### Effects of different plant extracts on fresh and dry weight of seedlings

The changes in fresh and dry weights of *M. truncatula* and alfalfa subjected to treatments with the two plant extracts (at a concentration of 6 mg/ml) were consistent, with both fresh and dry weights of the treated group significantly lower than those of the control group (Figures 4E,F). Among them, the fresh weight and dry weight of *M. truncatula* after treatment with alfalfa extracts were lower than those treated with *M. truncatula* extracts. After being treated with *M. truncatula* and alfalfa extracts, the fresh weight of *M. truncatula* decreased by 42.46 and 64.68% and the fresh weight of alfalfa decreased by 80.75 and 74.08%, respectively (Figure 4E). The changes in the dry weight showed the same trend (Figure 4F).

### Effects of different plant extracts on photosynthesis

**Figure 7:**
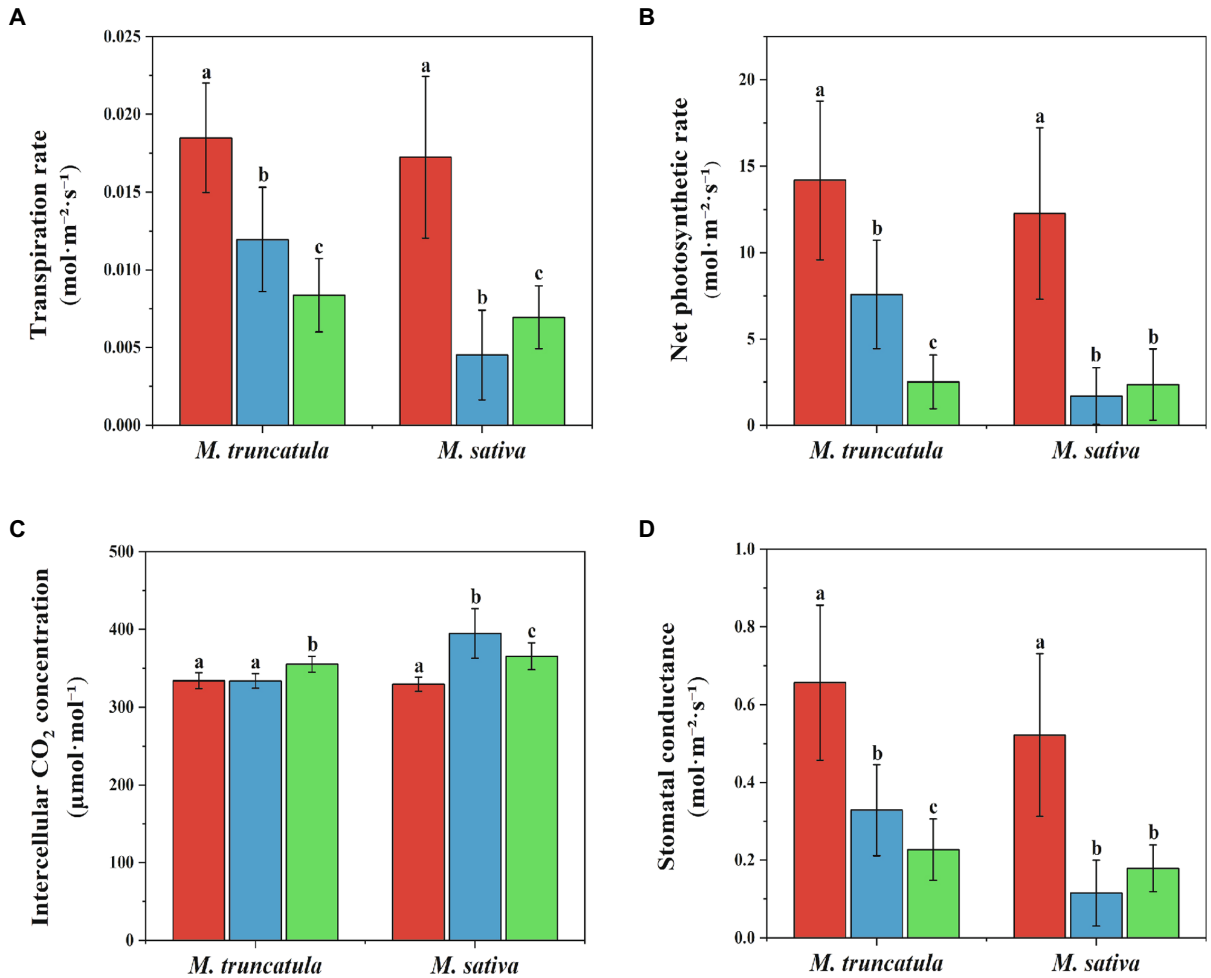
Effects of different plant extracts on photosynthesis during the growth of seedlings. **(A)** Transpiration rate; **(B)** net photosynthetic rate; **(C)** intercellular CO_2_ concentration; **(D)** stomatal conductance. Values represent means±SD of seven biological replicates. Different letters above the bars indicate significant difference (*p* < 0.05). 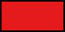 Control 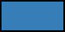 *M. truncatula* extracts 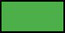 alfalfa extracts.

After being treated with *M. truncatula* and alfalfa extracts at a concentration of 6 mg/ml, the transpiration rate, net photosynthetic rate, and stomatal conductance of the treated group were significantly lower than those of the control group (Figure 7). The transpiration rate was reduced by 35.41 and 54.76% in *M. truncatula* and 73.80 and 59.65% in alfalfa (Figure 7A). The net photosynthetic rate decreased by 46.56 and 82.27% in *M. truncatula* and 86.08 and 80.72% in alfalfa (Figure 7B). Stomatal conductance was reduced by 49.97 and 65.42% in *M. truncatula* and 77.98 and 65.66% in alfalfa (Figure 7D).

## Discussion

Since it is believed that autotoxicity was caused by secondary metabolites with water solubility (Chung and Miller, 1995), this study selected two different sources of the autotoxic substances, one being root exudate of *M. truncatula* and the other being aqueous extracts of alfalfa and *M. truncatula* plants, to investigate their biological activities on *M. truncatula* and alfalfa as recipient plants. Since seed germination and seedling growth stages of a plant are sensitive to external environmental changes (Matias et al., 2014), and these two stages are often used to analyze allelopathy and autotoxicity effects (Ma et al., 2014; Huang et al., 2019; Kato-Noguchi and Kurniadie, 2020; Zhang et al., 2021b), this study also selected these two stages to investigate the autotoxic and allelopathic effects of *M. truncatula* and alfalfa. Since autotoxicity and allelopathy in *M. truncatula* are unknown, while autotoxicity in alfalfa is well-documented, we tested and analyzed the effects in both species.

We first tested the biological activities of *M. truncatula* root secretions on the species itself and found that the biomass decreased significantly with increasing concentrations, indicating a possibility of autotoxicity in *M. truncatula*. To confirm this observation, more detailed analyses with aqueous extracts of *M. truncatula* and alfalfa were carried out. Varying degrees of suppression were found with the extracts. At seed germination stage, extracts of *M. truncatula* not only suppressed the germination of *M. truncatula* and alfalfa seeds, but also drastically reduced the radicle length and hypocotyl length of these two closely related species. Similarly, extracts of alfalfa showed even more potent suppression effects on germination, radicle length, and hypocotyl length of both *M. truncatula* and alfalfa. As a direct receptor of plant extracts, root growth and development were inhibited by the effects of autotoxic stress. Such stress may result in reduced water and nutrient uptake and utilization by plants (Scavo et al., 2019), thus affects shoot development. Photosynthesis was also suppressed by autotoxic stress, which affects organic matter accumulation and leads to reduced plant height and biomass. These results demonstrate that *M. truncatula* has autotoxicity and show allelopathic effect on alfalfa.

To date, many chemical compounds potentially associated with autotoxicity and allelopathy have been isolated, the effects of these compounds are often characterized by *in vitro* analyses (Kato-Noguchi et al., 2018; Yan et al., 2019; Ma et al., 2020; Martins et al., 2021; Mousavi et al., 2021; Wang et al., 2021). However, it has not been possible to confirm the exact function of a certain compound by genetically knocking out its biosynthetic pathway (Cheng and Cheng, 2015). With the establishment of mutant populations and genomics tools in *M. truncatula*, it has become an important resource to answer some important basic biological questions, such as nitrogen fixation, compound leaf development, and seed physical dormancy (Chai et al., 2016; Kang et al., 2016; Mergaert et al., 2020; Zhao et al., 2021). These traits are not available in *Arabidopsis thaliana*. Now, this study shows that *M. truncatula* is also suitable for in-depth investigations of molecular and biochemical pathways of allelochemicals.

## Conclusion

Root exudates and plants extracts have negative effects on a number of traits related to germination and plant growth in the model legume *M. truncatula*. The current study proved, for the first time, that autotoxicity and allelopathic effects exist in *M. truncatula.* This opens up a new way of studying molecular and biochemical mechanisms behind autotoxicity and allelopathy.

## Data availability statement

The original contributions presented in the study are included in the article/supplementary material, further inquiries can be directed to the corresponding authors.

## Author contributions

Z-YW, CY, and JS designed the research. CW, ZL, ZW, WP, LZ, ZW, and YZ performed the experiments. CW, Z-YW, CY, and JS wrote the paper. All authors contributed to the article and approved the submitted version.

## Funding

This work was supported by the National Natural Science Foundation of China (U1906201) and the First Class Grassland Science Discipline Program of Shandong Province.

## Conflict of interest

The authors declare that the research was conducted in the absence of any commercial or financial relationships that could be construed as a potential conflict of interest.

## Publisher’s note

All claims expressed in this article are solely those of the authors and do not necessarily represent those of their affiliated organizations, or those of the publisher, the editors and the reviewers. Any product that may be evaluated in this article, or claim that may be made by its manufacturer, is not guaranteed or endorsed by the publisher.
